# The Role of V-ATPase ATP6V0D1 Subunit in Chemoresistance and Ellipticine-Induced Cytoplasmic Vacuolation in Neuroblastoma Cells

**DOI:** 10.1080/23723556.2025.2518774

**Published:** 2025-06-17

**Authors:** M Rychla, J Hrabeta, P Jencova, N Podhorska, T Eckschlager

**Affiliations:** Department of Pediatric Hematology and Oncology, 2nd Faculty of Medicine, Charles University and Motol University Hospital, Prague 5, Czech Republic

**Keywords:** Neuroblastoma, chemoresistance, V-ATPase, vacuolation

## Abstract

Drug resistance remains a major obstacle in neuroblastoma treatment. Lysosomal sequestration, facilitated by the V-ATPase proton pump, is one of the mechanisms of chemoresistance. Overexpression of the ATP6V0D1 subunit of V-ATPase, previously reported in various cancers, was also observed in ellipticine-resistant neuroblastoma cells in our study. Neuroblastoma cells also exhibited increased lysosomal capacity and vacuolation after ellipticine treatment. Knockdown of ATP6V0D1, but not ATP6V1H, enhanced ellipticine sensitivity, suppressed proliferation and migration, decreased lysosomal uptake, and induced G2/M arrest in neuroblastoma cell lines. Notably, inhibiting another V-ATPase subunit, ATP6V1H, had no effect, highlighting the specific role of ATP6V0D1 in drug resistance. Ellipticine-induced vacuolation, identified as endoplasmic reticulum swelling, lacked evidence of paraptosis. ATP6V0D1 knockdown suppressed this phenomenon, whereas ATP6V1H silencing did not. Our findings underscore the importance of ATP6V0D1 in neuroblastoma and suggest potential therapeutic strategies targeting V-ATPase for overcoming drug resistance.

## Introduction

Cancers belong among the most serious problems of modern medicine and their occurrence is constantly increasing. Despite the significant advancements in therapeutic methods over recent years, this disease remains difficult to treat.^1,2^ Neuroblastoma (NBL) is a malignant embryonal tumor arising from undifferentiated sympathetic cells. It represents the most frequent solid extracranial tumor in the pediatric population.^3,4^ NBL is characterized by heterogeneous behavior, where it can regress spontaneously (low-risk NBL), but it can also have aggressive behavior (high-risk NBL) and despite intensive induction therapy, only about 40% of patients have a complete response. NBL patients who have a poor response to induction therapy or relapse have a dismal outcome.^5–7^

Despite advances in the development of new treatment methods, the use of conventional cytostatics, such as doxorubicin, cisplatin and others, remains in most cases indispensable for the treatment of cancer, including NBL. However, it is known that antitumor treatment can induce resistance, which is associated with increased aggressiveness of tumor cells and disease progression.^8–10^ Ellipticine (5,11-dimethyl-6 h-pyrido[4,3-b] carbazole) and its derivatives are characterized by multiple biological activities but predominant mechanism of the antitumor effect is intercalation into DNA and inhibition of topoisomerase II. Ellipticines, including their novel derivatives, are currently being investigated for their potential to inhibit mutant p53 protein phosphorylation and induce DNA adduct complex.^11–13^

Resistance to cytostatic drugs is determined by a number of different mechanisms. One such mechanism is the vacuolar trapping of the hydrophobic weak base cytostatic drugs. Vacuolar ATPase (V-ATPase), a proton pump required for the acidification of vacuoles, plays an important role in vacuolar trapping as a sensor of cytosolic pH. Besides pH sensing this pump has also function in disease-related processes such as entry of toxins and viruses, membrane fusion and tumor cell invasiveness and angiogenesis.^14^ V-ATPase complex composed of fourteen subunits that form two sectors: an integral membrane-associated V0 sector and a cytosolic V1 sector capable of regulated dissociation from the V0 sector. The V1 sector, comprising subunits A to H, is responsible for adenosine triphosphate (ATP) hydrolysis. The V0 sector, composed of subunits a, d, e, c, and c″, forms the channel that conducts proton transport.^14,15^ Complete loss of V-ATPase activity is lethal early in mammalian development. However, mutations in individual subunits of human V-ATPase cause different phenotype manifestations. For example, mutations of the ATP6V0a1 isoform result in multiple neurological disorders, mutations in ATP6V0a2 are associated with autosomal recessive cutis laxa and loss of function mutations in ATP6V0a3 are associated with autosomal recessive osteopetrosis.^14,15^ Dysregulation of V-ATPase has been identified in many cancers e.g. elevated plasma membrane expression of V-ATPase was found in several highly invasive breast cancer cells and melanoma cells.^16–18^ V-ATPase subunits ATP6V1E and ATP6V0c, have been reported to correlate with cancer stage in pancreatic ductal adenocarcinoma, and ATP6V0D1 with osteosarcoma and specific V-ATPase subunits signature have been reported in glioma suggesting their potential role in tumor progression.^19–22^

The unique functions of individual V-ATPase isoforms underscore the importance of understanding their expression and potential dysregulation. Therefore, this study aims to characterize the expression profile of V-ATPase subunits in NBL cells, investigate their potential dysregulation, and elucidate their contribution to ellipticine chemoresistance and other biological processes. Our findings provide insights into the specific role of the ATP6V0D1 subunit of V-ATPase in mediating ellipticine resistance in NBL cells, highlighting its potential as a therapeutic target.

## Materials and methods

### Cell culture

UKF-NB-4, a human high-risk NBL cell line, was donated by prof. J. Cinatl from Goethe University in Frankfurt am Main. The cell line resistant to ellipticine UKF-NB-4^ELLI^, was prepared from chemosensitive parental cell line after long-term cultivation with increasing ellipticine concentration in our lab.^23^ Both cell lines were grown in Iscove’s Modified Dulbecco’s medium (IMDM, #12440061) supplemented with 10% (v/v) fetal bovine serum (#A3840402) (both Thermo Fisher Scientific) and incubated at 37°C in 5% CO_2_. For experiments, cells were treated with 5 µM ellipticine (Sigma-Aldrich, #CAS519–23–3) 24 hours after seeding. When using a specific V-ATPase inhibitor, cells were treated with 100 nM Bafilomycin A (Sigma-Aldrich, #SML1661) alone or in combination with ellipticine.

### Cell viability assay

Cell viability was assessed using either the MTT (3-(4,5-dimethylthiazol-2-yl) −2,5-diphenyltetrazolium bromide) or PrestoBlue assay. The cytotoxicity of ellipticine (ELLI) was determined via MTT assay, as detailed previously in Belhajova et al..^24^ To investigate impact of V-ATPase inhibition, cells were seeded in 24-well cell culture plate at a density of 4 × 10^4^ cells/well. Following treatment or silencing, cells were incubated with PrestoBlue Cell Viability Reagent (Thermo Fisher Scientific, #A13261) as recommended by the manufacturer, fluorescence intensity was determined (λex = 560 nm and λem = 590 nm) using a SpectraMax i3x Multi-Mode Microplate Reader (Molecular Devices). All samples were analyzed in triplicate.

### Measurement of lysotracker uptake

To determine the cell lysosomal capacity (volume) we measured fluorescence intensity of lysosome-specific dye Lysotracker Deep Red (LTR) (Thermo Fisher Scientific, #L12492). 10^4^ cells/well sensitive and resistant cells or cells after silencing were seeded in 96-well cell culture plate for 24 h. After treatment with 5 µM ellipticine, 100 nM bafilomycin A or their combination for 30 minutes, 50 nM LTR was added and cells were incubated for 30 min at 37 °C and washed with Hank’s Balanced Salt Solution (HBSS, Gibco, #14025092). LTR fluorescence intensity was measured by excitation wavelength of 541 nm and emission of 713 nm using imaging cytometer SpectraMax i3x Multi-Mode Microplate Reader (Molecular Devices). Each sample was analyzed in triplicate and three independent experiments were performed.

### RNA isolation and qRT-PCR

For qRT-PCR, we followed the methods previously described in Belhajova et al..^24^ PureLink RNA Mini Kit (Thermo Fisher Scientific, #12183018A) was used to isolate total RNA according to the manufacturer’s protocol. Reverse transcription was performed using gb Reverse Transcription Kit (Generi Biotech, #3012). Primers and probes hATP6V0D1_Q2, hATP6V0A1_Q1, hATP6V0E1_Q1, hATP6V0B_Q1, hATP6V0C_Q1, hATP6V1A_Q2, hATP6V1B2_Q1, hATP6V1C1_Q1, hATP6V1F_Q2, hATP6V1E2_Q1, hATP6V1D_Q1, hATP6V1H_Q2, hATP6V1G1_Q1 and POLR2A were designed and produced by Generi Biotech (Custom oligo synthesis, #1000–020). We chose *POLR2A* as a control gene, because it is homogeneously and uniformly expressed in NBL cells.^25–27^

### Transfection

To achieve targeted knockdown of V-ATPase subunits, cells were seeded onto culture dishes or plates and allowed to adhere for 24 hours. Transfection complexes were then prepared by diluting DharmaFECT transfection reagent (#T-2001–03) and the corresponding siRNA smart pools (ON-TARGETplus Human ATP6V0D1, #L-019238-02–0020; ATP6V1H, #L-010930-00–0010) or non-targeting siRNA control (#D-001320-01–20, all from Dharmacon) at a 1:49 ratio in serum-free IMDM. After a 5-minute incubation at room temperature, an equal volume of DharmaFECT was added to each siRNA solution, followed by a further 20-minute incubation. Complete IMDM medium (containing 10% FBS) was then added to the siRNA-DharmaFECT complexes at a 4:1 ratio. The original culture medium was removed, and cells were transfected with the prepared siRNA solutions. After 48 h cells were collected for further analysis and experiments.

### Western blot analysis

For western blot analysis, cells were treated with cytostatics, with 200 ng/ml tunicamycin (Cell signaling Technology, #12819), 50 nM MG132 (Sigma, #M7449) or transfected. Minor modifications were made to the method described in Belhajova et al..^24^ These included: (1) the use of PhosSTOP (Roche, #4906845001) for phosphorylated protein extraction, and (2) a 30-minute incubation of the membrane in guanidine solution with mercaptoethanol for the determination of polyubiquitinated proteins. Primary antibodies were used at the following dilutions: V-ATPase D1Mouse mAb (Santa Cruz Biotechnology, #sc -393,322) 1:500, V-ATPase H Mouse mAb (Santa Cruz Biotechnology, #sc -166,227), 1:500, Ubiquitin (Ubi-1) Mouse mAb (Cell Signaling Technology, #13–1600) 1:500, Calnexin Rabbit mAb (Cell Signaling Technology, #2679S) 1:1000. PERK Rabbit mAb (Cell Signaling Technology, #12185S) 1:1000, p-PERK Rabbit mAb (Invitrogen, #PA5–40294) 1:250, Alix Mouse mAb (Cell Signaling Technology, #2171S) 1:500. β-actin Mouse mAb (Sigma-Aldrich, #A1978) diluted 1:3000 was used as a loading control. Secondary antibodies Europium conjugated anti-IgG (Molecular Devices, mouse- #R8208, rabbit- #R8209) were diluted 1:5000.

### Cell proliferation

Cell proliferation was monitored in real-time using the xCELLigence RTCA DP Instrument (ACEA Biosciences Inc), as previously described by Belhajova et al .^24^

### Flow cytometry

Bip protein expression was assessed by flow cytometry. Briefly, cells were treated with 5 µM ellipticine, 200 ng/ml tunicamycin, and/or transfected with siRNA. Cells were then washed, trypsinized, fixed in 3.6% paraformaldehyde, and permeabilized with 90% methanol. Subsequently, cells were incubated with primary Bip Rabbit mAb diluted 1:50 (Cell Signaling Technology, #3177S), followed by Alexa Fluor® 647-conjugated secondary antibody (Thermo Fisher Scientific, #A21245) diluted 1:500. Fluorescence was measured on a BD FACSCelesta flow cytometer (BD Bioscience), and data were analyzed using FlowLogic software (Inivai Technologies). Cells incubated with secondary antibody only served as negative controls. Apoptosis was evaluated *via* detection of cleaved caspase-3. Following siRNA transfection and drug treatment, cells were fixed and permeabilized as described above, then stained with an Alexa Fluor 647–conjugated Cleaved Caspase-3 antibody (Cell Signaling Technology, #9602S) at a 1:50 dilution. After washing, samples were analyzed by flow cytometry and the percentage of cleaved caspase-3–positive cells was used to quantify apoptotic cell death.

### Cell cycle analysis

Following treatment, cells were fixed and stained with FxCycle™ Violet Ready Flow™ Reagent (Thermo Fisher Scientific, #R37166) as per the manufacturer’s instructions. Cell cycle distribution was subsequently analyzed by flow cytometry.

### Wound healing assay

Cell migration was evaluated using the wound healing assay under low serum conditions, as fully described in Belhajova et al.^24^ and Cormier et al..^28^

### Confocal microscopy

Cells were seeded in a 35 mm glass bottom dish at a density of 1.2 × 10^5^ cells/ml or 0.9 × 10^5^ cells/ml if cells were transfected. Cells were treated with 5 µM ellipticine for 4 hours. Subsequently, 25 nM lysosome-specific dye LTR (Thermo Fisher Scientific, #L12492), 1 µM endoplasmic reticulum-specific dye ER-Tracker Red (ETR) (Thermo Fisher Scientific, #34250) or 25 nM mitochondria labeling dye MitoTracker Deep Red FM for (Thermo Fisher Scientific, #M22426) for 30 min were added and subcellular localization of ellipticine and vacuole origin were monitored. For vacuolation monitoring, cells were treated with 10 µM BAPTA-AM (Thermo Fisher Scientific, #B1205) or 60 µM cycloheximide (Sigma, #01810 30 min prior to ellipticine treatment Images were acquired using the confocal microscope Leica SP8 (Leica Microsystems).

### Statistical analysis

All experiments were performed independently at least three times. Data are presented as mean ± standard error. Statistical comparisons were conducted using one-way ANOVA with Tukey’s HSD post-hoc test or two-way ANOVA followed by Bonferroni correction, as appropriate. Western blot and vacuole quantification were performed using ImageJ software. RT-qPCR data were analyzed using REST 2009 software.^29^ Statistical significance was defined as *p* < .05 and is indicated in the figures and corresponding legends.

## Results

### ATP6V0D1 subunit of V-ATPase is upregulated in ellipticine resistant neuroblastoma cell line

**Figure 1.**
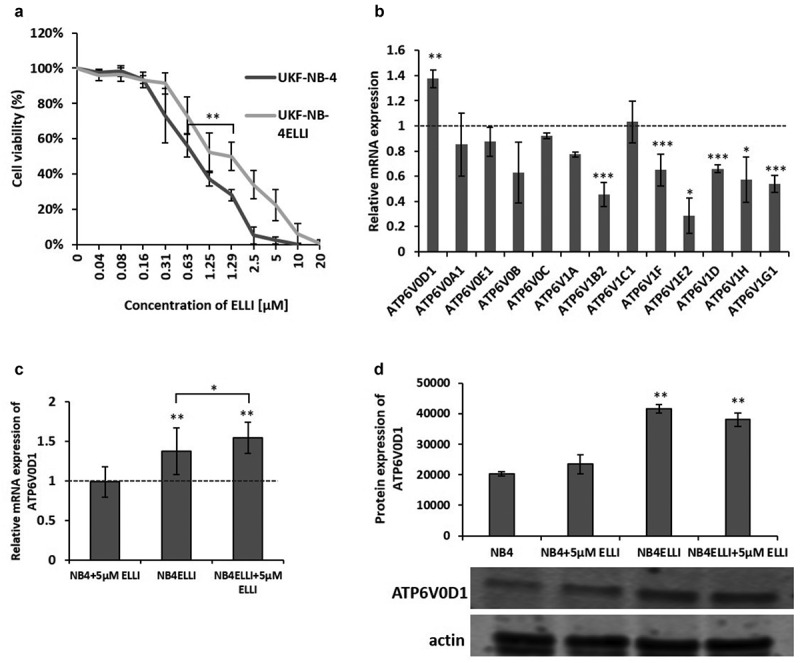
ATP6V0D1 subunit of V-ATPase is upregulated in resistant neuroblastoma cell line. MTT assay results show cytotoxicity of ellipticine (ELLI) after 48 h. UKF-NB-4 has IC_50_ 0.625 ± 0.037 µM and ELLI resistant UKF-NB-4^ELLI^ has IC_50_ 1.29 ± 0.1 µM (a). V-ATPase subunits expression of mRNA were determined by qRT-PCR. Results show significantly higher level of subunit ATP6V0D1 in ELLI resistant UKF-NB-4^ELLI^ cell line than in sensitive UKF-NB-4 cell line (*p* < .01) (b). Treatment with 5 µM ELLI for 24 h significantly increased ATP6V0D1 level in UKF-NB-4^ELLI^ (NB4ELLI) compared to UKF-NB-4 (NB4) (*p* < .05) (c). Expression of ATP6V0D1 protein detected by western blotting in UKF-NB-4 (NB4) and ELLI resistant UKF-NB-4^ELLI^ cell lines show same result as qRT-PCR. Actin was used as loading control. Quantification and representative western blot (d). Average and standard deviations (SD) from three independent experiments are shown. ***p < .001, **p < .01, *p < .05 (ANOVA with post-hoc Tukey HSD test).

Results from MTT assay confirm significantly lower sensitivity of ellipticine resistant NBL cell line, compared to the parental UKF-NB-4 cell line, with an IC_50_ approximately two-fold higher (UKF-NB-4- 0.625 ± 0.037 µM; UKF-NB-4^ELLI^-1.29 ± 0.1 µM) (Figure 1a). Analysis of mRNA levels in ellipticine-resistant cells revealed significant changes in the expression of several V-ATPase subunits. Notably, we observed a significant increase (*p* < .01) in the expression of ATP6V0D1, which encodes the d subunit of V-ATPase. Conversely, six subunits (V1B2, V1F, V1E2, V1D, V1H, and V1G1) were downregulated, while the expression of six other subunits (V0A1, V0E1, V0B, V0C, V1A, and V1C1) remained unchanged (Figure 1b). In resistant UKF-NB-4^ELLI^ cells, upregulation of ATP6V0D1 was also observed at the protein level (Figure 1d). Additionally, ellipticine treatment further increased ATP6V0D1 subunit expression in UKF-NB-4^ELLI^ cell line whereas this expression remained unaltered in sensitive cells (Figure 1c).

### Ellipticine resistant cell line shows higher lysosomal uptake and V-ATPase inhibition potentiates the effects of ellipticine

**Figure 2.**
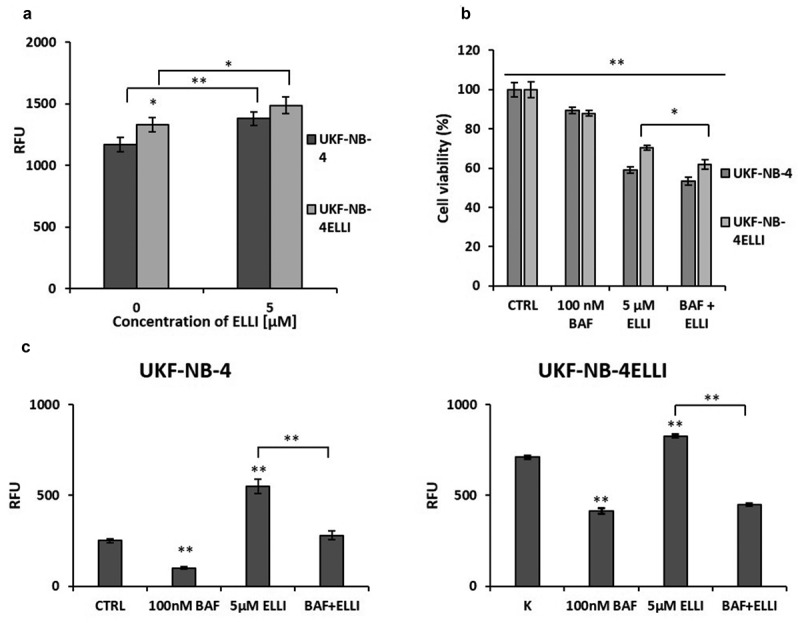
Ellipticine resistant cell line shows higher lysosomal capacity and V-ATPase inhibition potentiates the effects of this anticancer agent. NBL cells incubated with 200 nM lysosome-specific dye LTR showed increased fluorescence intensity of LTR in ELLI resistant cell line UKF-NB-4^ELLI^ compared to sensitive cell line UKF-NB-4 (*p* < .05). Treatment of NBL cell with 5 µM ellipticine (ELLI) increased fluorescence intensity of LTR in both sensitive and resistant cells (a). Cells were treated with 100 nM bafilomycin, 5 µM ellipticine or their combination for 24 hours. Co-treatment with ellipticine and bafilomycin resulted in lower viability in resistant cell line UKF-NB-4^ELLI^ compared to treatment with ellipticine alone (b). Treatment of cells with 100 nM V-ATPase inhibitor bafilomycin a (BAF) decreased fluorescence intensity of LTR (c) in NBL cells in both lines and reduced ellipticine-induced increase of LTR fluorescence. Average and standard deviations (SD) from three independent experiments are shown. **p < .01, *p < .05 (ANOVA with post-hoc Tukey HSD test).

As we previously demonstrated, ellipticine localizes to lysosomes.^30^ To investigate lysosomal capacity, we used lysosome-specific dye LTR. Results show significantly higher LTR fluorescence intensity in the resistant cell line (Figure 2a). Ellipticine increased the uptake of LTR into lysosomes, suggesting an increase in V-ATPase activity (Figure 2a). To confirm this, cells were treated with V-ATPase inhibitor bafilomycin A, either alone or in combination with ellipticine. Bafilomycin treatment led to a reduction in LTR fluorescence intensity and decreased ellipticine-induced increase of lysosomal capacity in both cell lines (Figure 2c). Furthermore, co-treatment with bafilomycin significantly enhanced the sensitivity of the resistant UKF-NB-4^ELLI^ cell line to ellipticine after 24-hour exposure, as demonstrated by a viability assay (*p* < .05, Figure 2b).

### ATP6V0D1 knockdown potentiates ellipticine cytotoxicity and inhibits cell proliferation

**Figure 3.**
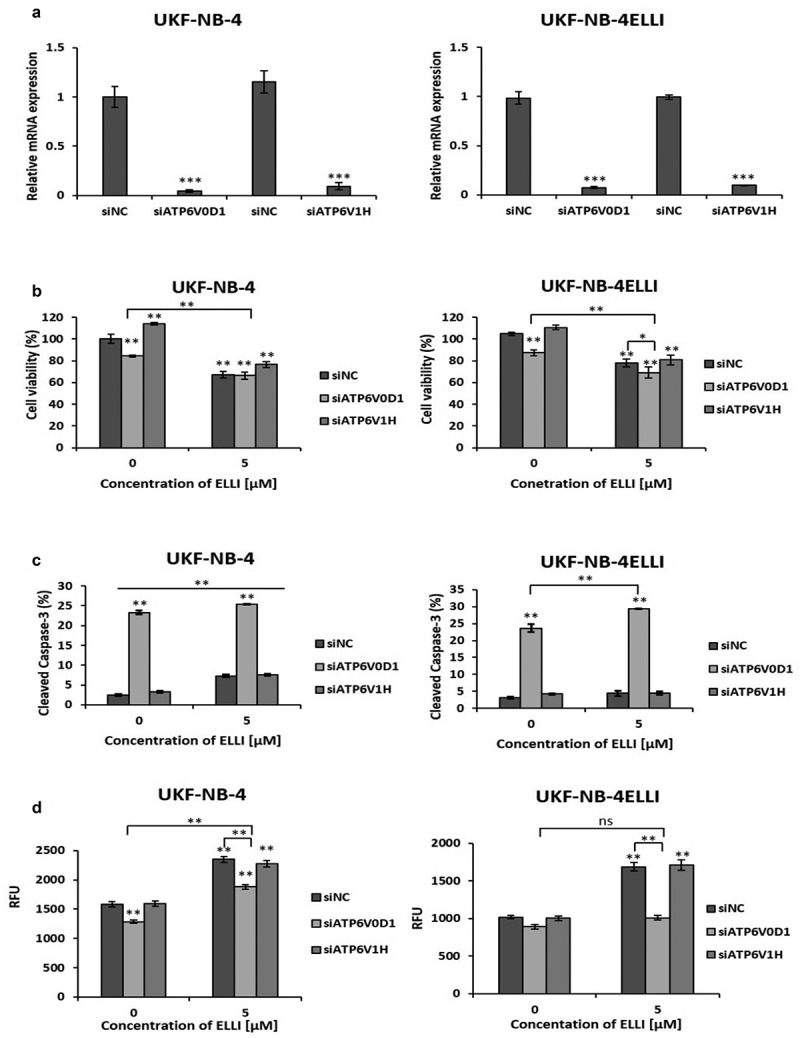
ATP6V0D1 knockdown potentiates ellipticine cytotoxicity. qRT-PCR showed decrease in expression of ATP6V0D1 and ATP6V1H in UKF-NB-4 and UKF-NB-4^ELLI^ cells transfected with siATP6V0D1, siATP6V1H, non-coding siRNA (siNC) was used as a control (a). Results from viability assay after ATP6V0D1 and ATP6V1H downregulation and treatment with 5 µM ELLI (b). Apoptosis assessed by cleaved caspase-3, is significantly elevated in cells transfected with siATP6V0D1 (c). Knockdown of ATP6V0D1 decreased fluorescence intensity of LTR (d) in NBL cells and in both lines treated with ellipticine. Average and standard deviations (SD) from three independent experiments are shown. ***p < .001, **p < .01(ANOVA with post-hoc Tukey HSD test).

Since bafilomycin-mediated inhibition of V-ATPase increased sensitivity to ellipticine in resistant UKF-NB-4^ELLI^ cell line, we investigated whether ATP6V0D1 subunit is associated with chemoresistance. We transfected NBL cells with ATP6V0D1 siRNA for 48 hours. In parallel, ATP6V1H subunit was also silenced for comparative analysis. Transfection resulted in a significant suppression of both V-ATPase subunits (*p* < .001) on mRNA and protein level (Figure 3a, S1). Cell viability assays conducted 48 hours post-transfection revealed a significant reduction in viable cells following ATP6V0D1 silencing in both NBL cell lines. In contrast, siATP6V1H had no significant impact on cell viability (Figure 3b). Subsequent treatment with ellipticine significantly decreased the viability of resistant UKF-NB-4^ELLI^ cells transfected with ATP6V0D1 siRNA, whereas the viability of the parental sensitive cell line remained unchanged compared to the treated control (siNC) (Figure 3b). Consistent with these findings, measurement of cleaved caspase-3 levels demonstrated that knockdown of the ATP6V0D1 subunit led to a marked increase in active caspase-3 in both cell lines, with the highest levels observed in combination with ellipticine treatment (Figure 3c), indicating enhanced apoptosis. Knockdown of ATP6V0D1 subunit significantly inhibited cell proliferation, as evaluated by the xCELLigence system, and these cells were most sensitive to ellipticine treatment. In contrast, knockdown of ATP6V1H had no effect on viability, levels of cleaved caspase-3, proliferation and sensitivity to ellipticine (Figure 3c, 4a,b). Furthermore, consistent with the effects of bafilomycin treatment, knockdown of the ATP6V0D1 subunit resulted in a decrease in LTR fluorescence intensity in UKF- NB-4 cells, both with and without ellipticine treatment. In the resistant UKF-NB-4^ELLI^ cell line, there was no significant reduction of LTR fluorescence. However, silencing of ATP6V0D1 maintained LTR fluorescence intensity at levels comparable to the untreated control, even following ellipticine exposure. Knockdown of the ATP6V1H subunit did not affect LTR fluorescence compared to the control in either NBL cell lines (Figure 3d).

**Figure 4.**
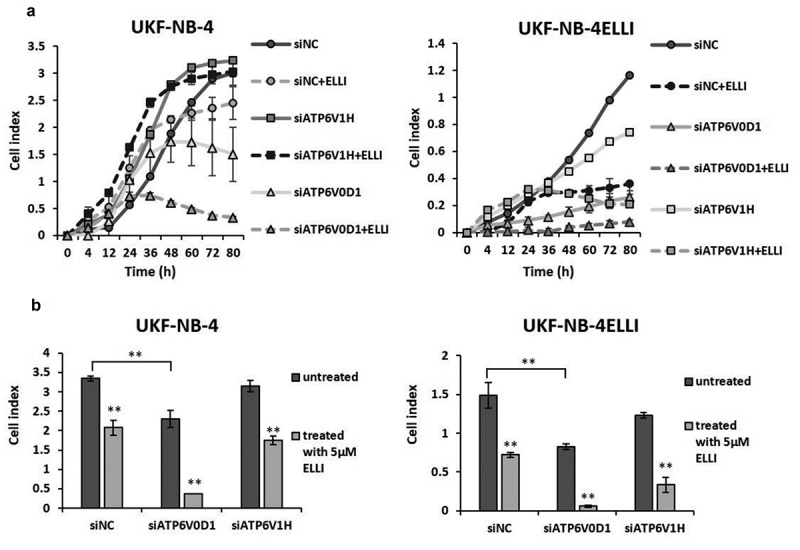
ATP6V0D1 knockdown inhibits cell proliferation. Proliferation assayed via xCelligence system after ATP6V0D1 and ATP6V1H downregulation and treatment with 5 µM ELLI, representative data for UKF-NB-4 and UKF-NB-4^ELLI^ (a). Bar chart shows quantification of cell index after 72 hours (b). Average and standard deviations (SD) from three independent experiments are shown. * *p* < .05 (ANOVA with post-hoc Tukey HSD test).

### ATP6V0D1 knockdown inhibits neuroblastoma cell migration and causes cell cycle arrest

**Figure 5.**
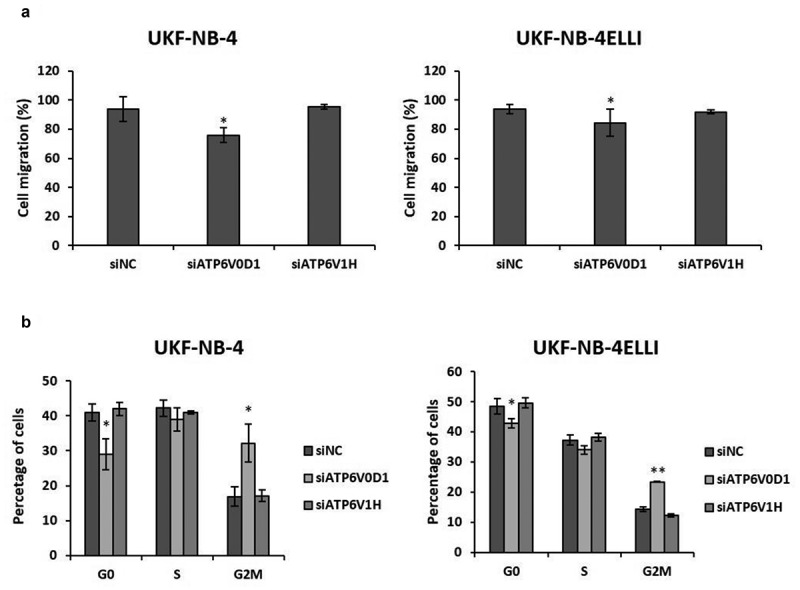
ATP6V0D1 knockdown inhibits neuroblastoma migration and induces arrest of cells in G_2_/M phase in both the UKF-NB-4 and UKF-NB-4^ELLI^ cells. The result of wound healing assay, statistical chart for the scratch-wound gap after 72 h of UKF-NB-4 and UKF-NB-4^ELLI^ cells after ATP6V0D1 and ATP6V1H downregulation (a). Results of cell cycle analysis of UKF-NB-4 and UKF-NB-4^ELLI^ cells after ATP6V0D1 and ATP6V1H downregulation (b). Average and standard deviations (SD) from three independent experiments are shown * *p* < .05 (ANOVA with post-hoc Tukey HSD test).

Since downregulation of ATP6V0D1 subunit of V-ATPase inhibited cell proliferation, we explored the impact of ATP6V0D1 knockdown on cell cycle and migration. Consistent with proliferation assay data, the wound healing assay showed significantly decreased cell migration after knockdown of the ATP6V0D1 subunit, whereas knockdown of the ATP6V1H subunit had no effect in both sensitive and resistant NBL cell lines (Figure 5a, S2). Flow cytometry analysis of cell cycle distribution revealed a significant increase in proportion of cells in G_2_/M phase in both the UKF-NB-4 and UKF-NB-4^ELLI^ cell lines after ATP6V0D1 siRNA transfection as compared to the control group (Figure 5b). ATP6V1H silencing did not lead to any significant change in cell cycle (Figure 5b).

### Downregulation of ATP6V0D1 diminished ellipticine-induced vacuolation in NBL cells

**Figure 6.**
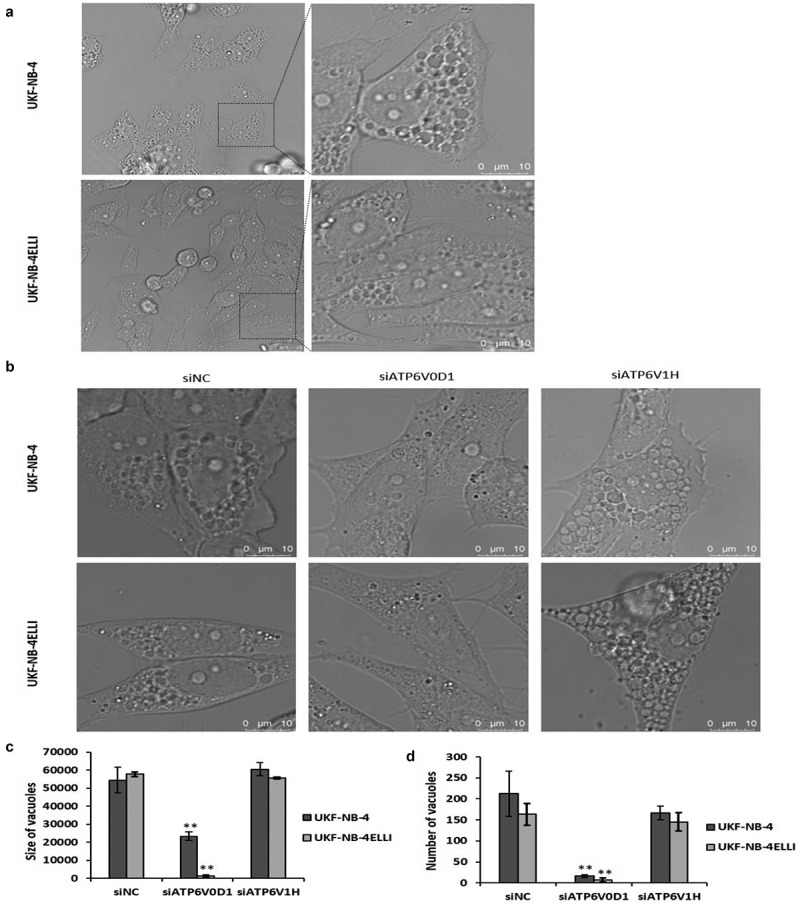
Ellipticine induced vacuolation is diminished by ATP6V0D1 knockdown. Confocal microscope images show vacuolation in UKF-NB-4 and UKF-NB-4^ELLI^ after 4 h treatment with 5 µM ellipticine (a). Vacuoles are present also after ATP6V1H downregulation (siATP6V1H) and in control (siNC) after 4 h treatment with 5 µM ellipticine. After ATP6V0D1 downregulation no vacuoles were observed (b). Quantitative analysis shows a reduced size (c) and number (d) of vacuoles following ATP6V0D1 knockdown. Representative data from one of three independent experiments are shown, magnifications 630x.

**Figure 7.**
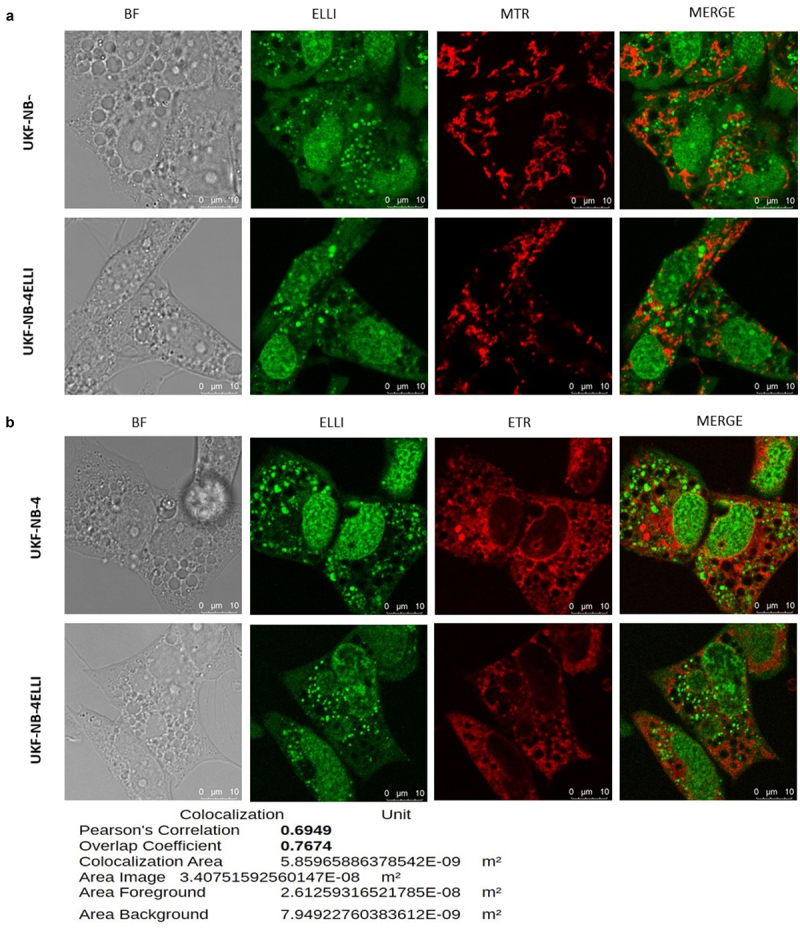
Confocal microscope images of subcellular localization of 5 µM ellipticine (green) after 4 h in UKF-NB-4 and UKF-NB-4^ELLI^ cells. Ellipticine do not co-localize with Mito tracker (MTR) and is outside of vacuoles. Fluorescent staining of mitochondria by Mito tracker (red) show the ellipticine-induced vacuoles were not derived from mitochondria (a). Subcellular localization of 5 µM ellipticine (green) after 4 h in UKF-NB-4 and UKF-NB-4^ELLI^ cells. Ellipticine do not co-localize with ER tracker (ETR) and is outside of vacuoles. Fluorescent staining of endoplasmic reticulum by ER tracker (red) suggested the ellipticine-induced vacuoles were derived from ER (b). Representative data from one of three independent experiments is shown, magnifications 630x. BF- bright field.

Based on the previously described entry of ellipticine into lysosomes and its associated vacuolation, we investigated the impact of silencing ATP6V0D1 subunit on this process.^31^ Ellipticine, a weak base, preferentially accumulates in lysosomes due to the pH gradient. Its inherent fluorescence allows direct visualization of its subcellular localization. Interestingly, ellipticine induces massive cytoplasmic vacuolation, which was evident as early as 30 min after the initiation of treatment with ellipticine (Figure 6a, S3). Knock down of ATP6V0D1, but not ATP6V1H, significantly suppressed vacuole size and number (Figure 6b,c,d). To determine the origin of these vacuoles, we used mitochondrial stain Mitotracker (MTR), endoplasmic reticulum stain ETR and lysosomal stain LTR. In both neuroblastoma cell lines, strong co-localization of ellipticine with the lysosomal marker LysoTracker Red is evident in the merged images, as indicated by the yellow signal resulting from the red-green overlay (Fig. S4). These findings support the hypothesis that V-ATPase-mediated acidification facilitates the lysosomal sequestration of ellipticine. Upon treatment with the specific V-ATPase inhibitor bafilomycin A1 (BAF), lysosomal structures were almost completely depleted (Fig. S5), resulting in a pronounced redistribution of ellipticine, which accumulated predominantly in the nuclei. The merged images in Figure 7a show minimal overlap between vacuoles and MTR signals, indicating that ellipticine-induced vacuoles do not originate from mitochondria, but instead showed co-localization with the endoplasmic reticulum (ER) marker ER-Tracker Red, suggesting that these vacuoles originate from the ER (Figure 7b).

### Ca^2+^ chelator BAPTA prevents ellipticine-induced vacuole formation

**Figure 8.**
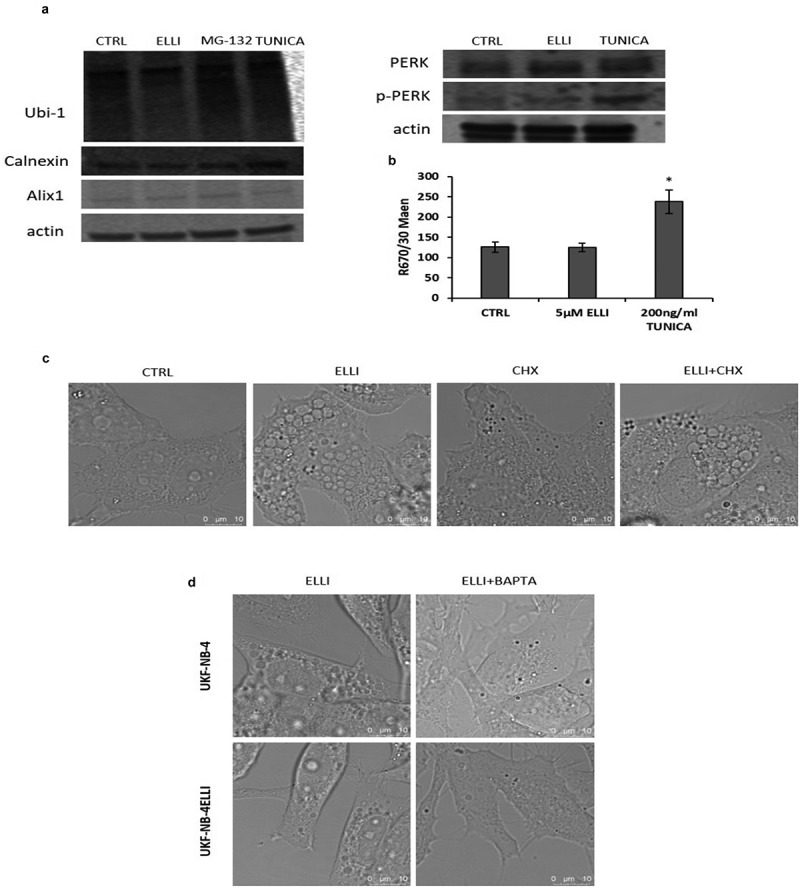
Ellipticine does not activate UPR. Results from western blot show no change in polyubiquitinated proteins (Ubi-1) and expression of celnexin, Alix1 and p-PERK after 24 h treatment with 5 µM ellipticine in UKF-NB-4 cells. Cells treated with Tunicamycin (200 ng/ml, TUNICA) were used as a positive control of UPR and 50 nM MG-132 was positive control for polyubiquitinated proteins (a). Higher expression of BiP protein after Tunicamycin (TUNICA) treatment was detected by flow cytometry. No change after ellipticine (ELLI) treatment was observed compared to control (CTRL) (b). Confocal images of UKF-NB-4 cells after treatment with 5 µM ellipticine (ELLI), 60 µM cycloheximide (CHX) or their combination (ELLI+CHX) for 4 h. Untreated cells were used as control (CTRL). Pretreatment of cells with CXH has no effect on vacuolation (c). Confocal images, pretreatment of UKF-NB-4 and UKF-NB-4^ELLI^ cells with 10 µM BAPTA (ELLI+BAPTA) stopped formation of vacuoles (d). Average and standard deviations (SD) from three independent experiments are shown. * *p* < .05 (ANOVA with post-hoc Tukey HSD test).

ER swelling due to ER stress has been reported to be associated with a phenomenon called paraptosis. Therefore, we investigated whether ellipticine induces this form of programmed cell death. Paraptosis is associated with accumulation of newly synthesized, misfolded proteins, leading to activation of Unfolded Protein Response (UPR) to restore ER function. During UPR chaperone protein BiP is upregulated. The Protein Kinase R-like ER Kinase (PERK) initiates its pathway by autophosphorylation (pPERK), which results in the halting of translation initiation. Calcium-binding protein Alix plays an inhibitory role under normal conditions and is downregulated during paraptosis.^32–35^ Flow cytometry analysis shows an increase in BiP protein expression after Tunicamycin treatment, a known inducer of ER stress, but not after ellipticine treatment (Figure 8b). Western blot analysis revealed that ellipticine did not modulate the expression of Alix, Calnexin, or p-Peark, nor did it increase the levels of polyubiquitinated proteins (Figure 8a). Furthermore, inhibition of protein synthesis by cycloheximide did not prevent the ellipticine induced cytoplasmic vacuolation (Figure 8c), therefore UPR is probably not involved. However, pretreatment of NBL cell with BAPTA, a cell-permeant calcium chelator, inhibited ellipticine induced vacuolation, thereby suggesting that Ca^2+^ might be involved in this process (Figure 8d).

## Discussion

NBL, a childhood cancer with poor outcomes, highlights the urgent need for innovative and more effective cancer treatments. Despite advancements in oncology, drug resistance remains a significant challenge, contributing to the difficulty in treating these diseases effectively. One mechanism contributing to drug resistance is lysosomal sequestration. In this process, certain drugs, particularly weak bases with hydrophobic properties, accumulate within lysosomes, cellular organelles with an acidic environment maintained by the V-ATPase proton pump. This sequestration impedes the drugs from reaching their intended cellular targets, thereby rendering them ineffective.^30,36,37^ Overexpression of certain V-ATPase subunits has been observed in various cancer types, including breast, lung, and pancreatic cancer.^19,38–41^ In addition, V-ATPase subunit composition has also been shown to correlate with aggressiveness in patient-derived glioblastoma cells and that glioblastoma and recurrent gliomas have a specific V-ATPase signature.^21^ Our findings indicate that the ATP6V0D1 subunit of V-ATPase is overexpressed in ellipticine-resistant NBL cell line, which also shows altered expression of other V-ATPase subunits. This observation is consistent with our previous study, which demonstrated overexpression of the V-ATPase d subunit in ellipticine- and cisplatin-resistant neuroblastoma cell lines.^31,42^ V-ATPase, beyond its role as a proton pump, regulates key signaling pathways implicated in cancer progression. It controls the trafficking and recycling of receptor tyrosine kinases (RTKs) like EGFR and HER2, impacting cell proliferation, survival, and migration.^43,44^ Its activity also influences mTOR signaling by modulating amino acid sensing and lysosomal functions, crucial for cell growth and metabolism.^45^ Additionally, V-ATPase regulates the acidification of endosomes, necessary for activating the Wnt signaling pathway, often dysregulated in cancer. Furthermore, V-ATPase is essential for autophagy, a process degrading damaged cellular components, which can either promote or suppress tumor growth depending on the context. This proton pump may also influence other signaling pathways involved in cancer cell proliferation, survival, and migration, such as the MAPK and PI3K/Akt pathways.^46^ Inhibition of V-ATPase has been shown to significantly reduce the growth, migration and invasion of cancer cells.^47^ Combining chemotherapy drugs with V-ATPase inhibitors yields a synergistic anticancer effect in drug-resistant cancer cells exhibiting heightened V-ATPase activity. In cisplatin-resistant ovarian cancer, overexpression of the a2 V-ATPase subunit is observed, and its inhibition induces apoptosis by interfering with DNA repair, and restores cisplatin sensitivity. Similarly, high expression of the V-ATPase C1 subunit correlates with multidrug resistance (MDR) in oral squamous cell carcinoma.^48,49^ Our study confirms these findings, demonstrating increased ellipticine sensitivity upon bafilomycin inhibition or knockdown of the V-ATPase d subunit (ATP6V0D1) in ellipticine resistant NBL cells. In addition to its effects on cell viability, silencing of the ATP6V0D1 subunit of the V-ATPase complex significantly impaired NBL cell proliferation and migration, and induced cell cycle arrest in the G2/M phase. The observed G2/M accumulation following ATP6V0D1 knockdown indicates a critical role for the V0D1 subunit in regulating cell cycle progression. One potential mechanism involves the disruption of intracellular pH homeostasis and lysosomal acidification- processes that are tightly controlled by V-ATPase activity. Proper acidification of intracellular compartments is essential for the degradation of cell cycle regulators and the functioning of key signaling pathways, including those mediated by cyclin B1 and CDK1, which orchestrate the G2/M transition. Inhibition of V-ATPase activity has been shown to impair lysosomal degradation and autophagic flux, leading to the accumulation of dysfunctional proteins and organelles that can activate DNA damage responses and checkpoint pathways, ultimately resulting in cell cycle arrest.^46,50^ Moreover, knockdown of ATP6V0D1 may interfere with the structural integrity of mitotic spindles or centrosomes, thereby impairing mitotic entry and progression. This is supported by evidence that V-ATPase contributes to centrosome positioning and spindle orientation.^51^ He, J. et al. demonstrated that the d subunit of V-ATPase plays a crucial role in MDR development, exemplified by its positive regulation of yes-associated protein (YAP), contributing to paclitaxel resistance in epithelial ovarian cancer and proton pump inhibitor esomeprazole significantly enhances drug sensitivity in these cells.^52^ In addition, ATP6V0D1 expression was higher in patients with stage III-IV ovarian cancer than in stage I-II ovarian cancer.^52^ V-ATPase facilitates the accumulation of ellipticine within the lysosome and this phenomenon is accompanied by massive vacuolation,^31^ this is consistent with our observations of increased lysosomal capacity (LTR fluorescence intensity) in resistant cells upon treatment. Notably, inhibiting with Bafilomycin A or silencing V-ATPased subunit led to a reduction in LTR fluorescence intensity and knockdown of d subunit diminished these ellipticine induced vacuoles. Our results suggest that the newly formed vacuoles are derived from the ER. ER swelling is a morphological change observed in response to various cellular stressors. To cope with this stress, cells initiate an adaptive response known as the unfolded protein response (UPR). The UPR is triggered by the activation of three ER transmembrane proteins: Inositol Requiring Enzyme 1 (IRE1), PKR-like ER kinase (PERK), and Activating Transcription Factor 6 (ATF6).^53^ However, we did not confirm UPR involvement in ellipticine-mediated vacuolation. Hägg, M. et al. obtained similar results, confirming that different ellipticine analog 6-propanamine ellipticine induce increased expression of the GRP78/BiP chaperone in response to ER stress in MDA-MB-231 breast carcinoma cells.^54^ ER swelling can occur due to calcium dysregulation. Given that lysosomes are known to be small stores of Ca^2+^, there may be a connection between lysosomal calcium handling and ER swelling.^53,55^ Treatment of NBL cells with BAPTA, a calcium chelator, inhibited ellipticine-mediated vacuole formation, thus confirming the involvement of calcium in this process. The mechanism by which calcium contributes to ellipticine-induced vacuole formation remains unclear, but it may involve TRPML1, a lysosomal Ca^2+^ channel that plays a key role in Ca^2+^ release from lysosomes. TRPML1-mediated Ca^2+^ release triggers various Ca^2+^-dependent processes, including lysosome biogenesis and autophagy. Notably, TRPML1 activation is dependent on lysosomal acidification, which is maintained by V-ATPase. Inhibition of V-ATPase by Bafilomycin A1 disrupts lysosomal acidification and, consequently, blocks TRPML1 activation, further supporting a potential role for TRPML1 in calcium-dependent vacuole formation.^55,56^

In this study, we confirmed the overexpression of the V-ATPase subunit ATP6V0D1 in a NBL cell line resistant to ellipticine, which also exhibited increased lysosomal capacity, as evidenced by enhanced LysoTracker (LTR) uptake. Upregulation of ATP6V0D1 was also observed in cisplatin-resistant NBL cell lines UKF-NB-4^CDDP^ and UKF-NB-2^CDDP^ (data not shown). Ellipticine-induced vacuolation was evident in both cell lines and may represent a protective cellular mechanism, as knockdown of ATP6V0D1 markedly reduced vacuole formation. The precise molecular mechanism underlying this vacuolation remains to be elucidated. Importantly, inhibition of the ATP6V0D1 subunit significantly increased the sensitivity of resistant cells to ellipticine, suppressed cell proliferation and migration, decreased lysosomal activity (LTR uptake), and induced G2/M cell cycle arrest in both NBL cell lines. In contrast, inhibition of another V-ATPase subunit, ATP6V1H, had no detectable effect on these cellular processes, highlighting the subunit-specific role of ATP6V0D1.

The findings of this study have important clinical implications for the treatment of high-risk and chemoresistant NBL. The identification of ATP6V0D1 as a critical mediator of ellipticine resistance suggests that this V-ATPase subunit could serve as a promising therapeutic target. Since V-ATPase activity influences various cancer hallmarks, including proliferation, migration, and resistance targeting specific subunits such as ATP6V0D1 could enhance the efficacy of conventional chemotherapy. Pharmacologic inhibitors of V-ATPase, such as bafilomycin A1, showed synergistic effects with ellipticine *in vitro*, indicating that combination strategies may overcome drug resistance. Moreover, selective silencing of ATP6V0D1, but not ATP6V1H, suppressed proliferation and migration, underscoring the subunit-specific vulnerability of NBL cells. These findings lay the groundwork for developing subunit-specific inhibitors that could be more effective and less toxic than global V-ATPase inhibition. Future directions could involve exploring ATP6V0D1 expression as a biomarker for chemoresistance or treatment response in patient-derived samples. Additionally, these mechanistic insights could inform the development of nanocarrier-based drug delivery systems that exploit lysosomal sequestration dynamics or pH gradients within tumors.

Despite the promising *in vitro* results, this study has several limitations. Most notably, all experiments were conducted using cultured NBL cell lines, and the findings have not yet been validated *in vivo*. While *in vitro* models are essential for elucidating molecular mechanisms, they cannot fully replicate the complexity of the tumor microenvironment or drug responses and metabolism. The absence of *in vivo* validation limits the ability to assess therapeutic efficacy and potential toxicity in a whole-organism context. Future studies employing patient-derived xenograft models will be essential to confirm the role of ATP6V0D1 in chemoresistance and tumor progression under physiologically relevant conditions. Such models will also enable evaluation of the therapeutic potential of ATP6V0D1 inhibition, using both genetic and pharmacological approaches, in a clinically relevant setting.

## Supplementary Material

supplemetray data clean.docx

## Data Availability

The datasets generated and/or analyzed during the present study are available from the corresponding author upon reasonable request.
