# Supplementary material for: The Role of V-ATPase ATP6V0D1 Subunit in Chemoresistance and Ellipticine-Induced Cytoplasmic Vacuolation in Neuroblastoma Cells
Source: Mol Cell Oncol. 2025 Jun 17;12(1):2518774. doi: 10.1080/23723556.2025.2518774 (PMC12184147; doi:10.1080/23723556.2025.2518774)
Supplement: supplemetray data clean.docx [file KMCO_A_2518774_SM8091.docx]

Supplemetray data


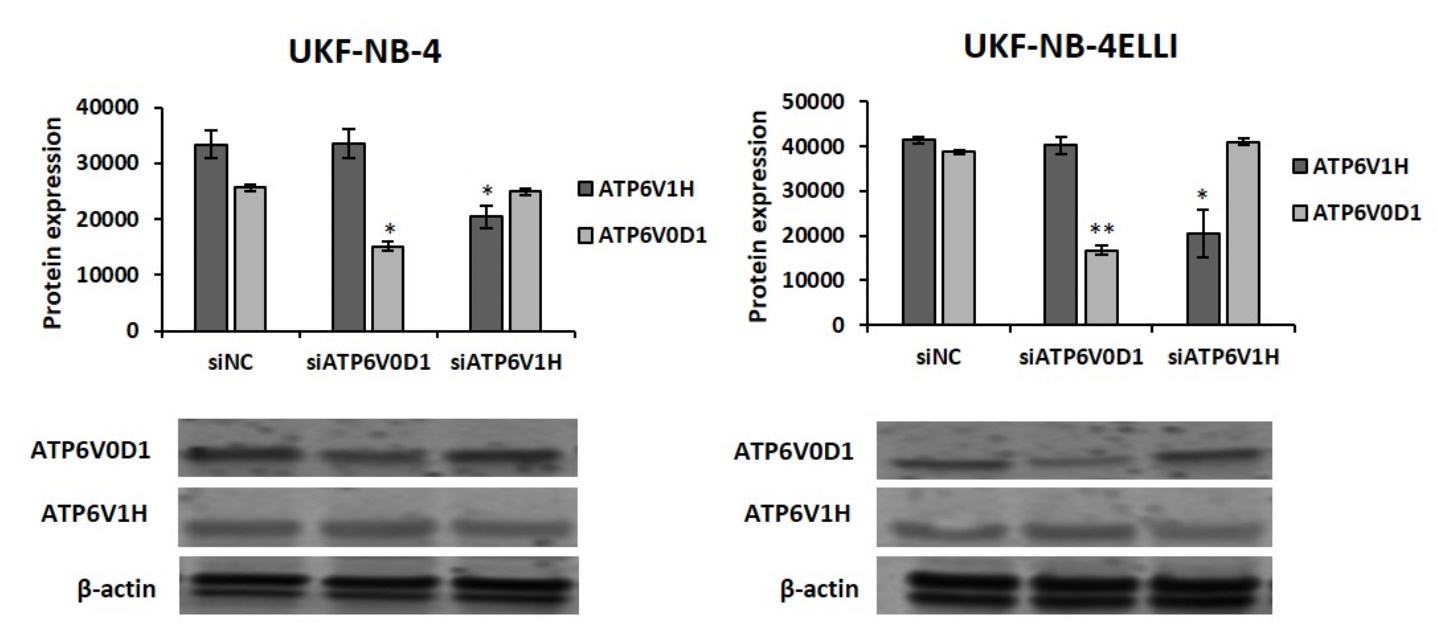


**Figure S1. Western blot** showed decrease in expression of ATP6V0D1 *and* ATP6V1H on protein level in UKF-NB-4 and UKF-NB-4^ELLI^ cells transfected with siATP6V0D1, siATP6V1H, non-coding siRNA (siNC) was used as a control.


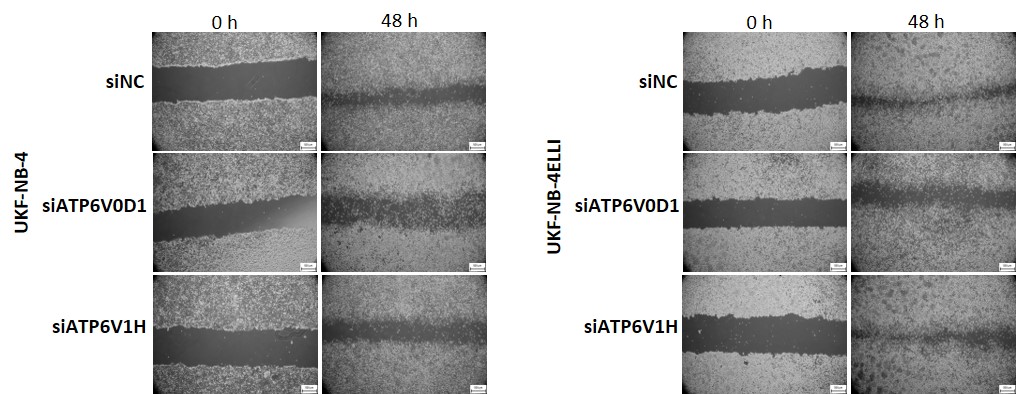


**Figure S2. ATP6V0D1 knockdown inhibits neuroblastoma migration.** The result of wound healing assay in UKF-NB-4 and UKF-NB-4^ELLI^ cells after ATP6V0D1 and ATP6V1H downregulation, showing decreased cell migration after ATP6V0D1 knockdown. Representative images taken immediately and 48 hours after the wounding.


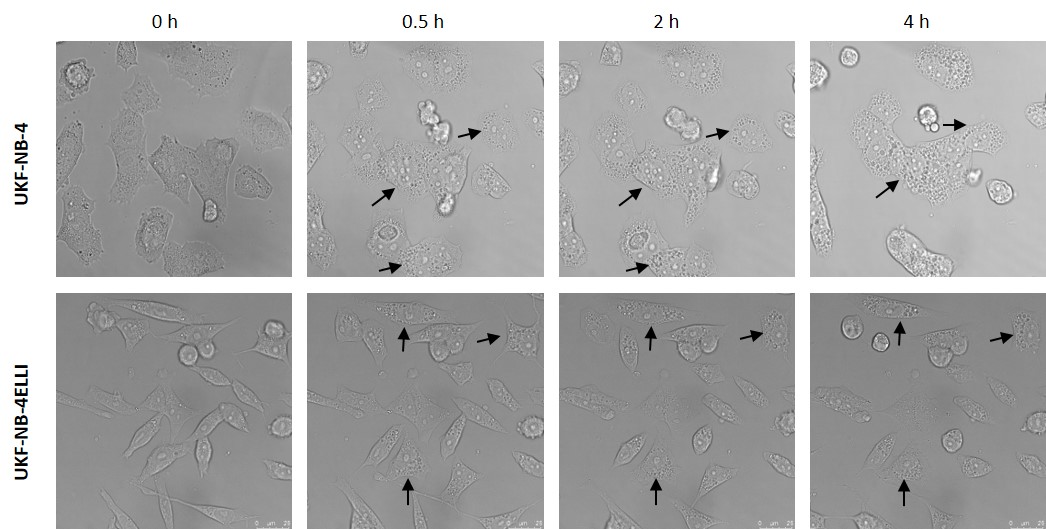


**Figure S3.** **Ellipticine induced vacuolization in the UKF-NB4 and UKF-NB-4^ELLI^ neuroblastoma cell line.** Representative images taken at different time points. Confocal images, TDR, magnifications 630x


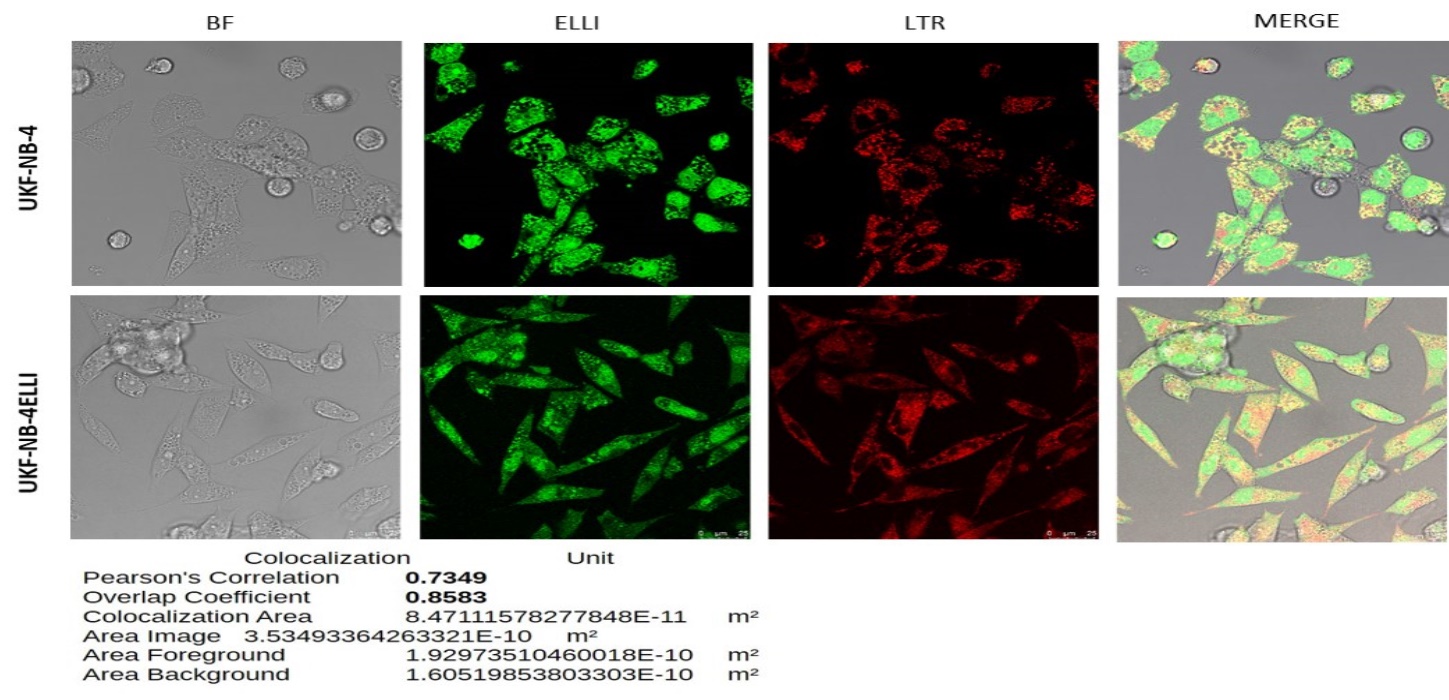


**Figure S4.** **Confocal microscope** images demonstrate co-localization (yellow) of ellipticine (green) and LysoTracker (red), (marker of the acidic lysosomal compartment) in UKF-NB-4 and UKF-NB-4^ELLI^ cells. This indicates that ellipticine is present (sequestrated) in lysosomes. Representative data from one of three independent experiments is shown. Confocal images, TDR, magnifications 630x


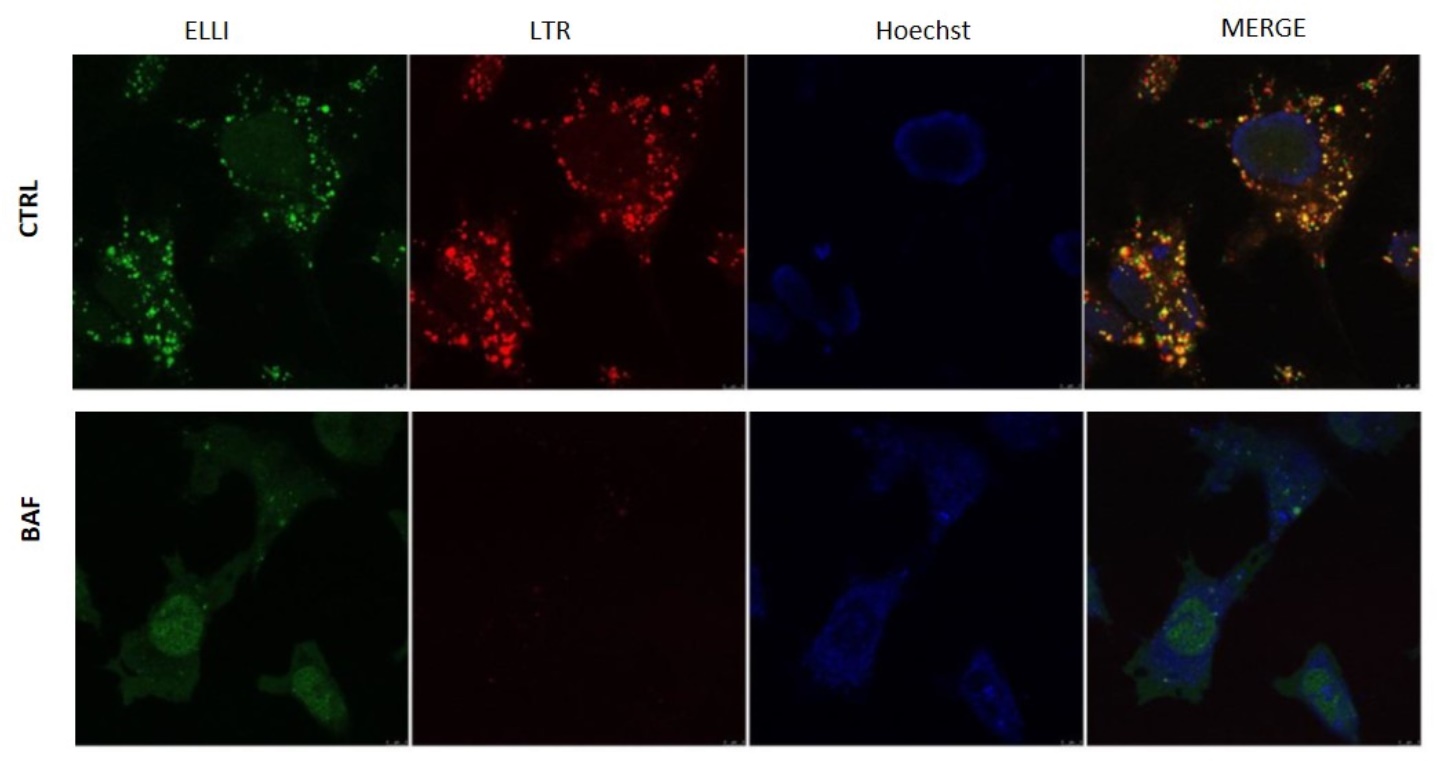


**Figure S5.** **Localization of ellipticine** in UKF-NB-4 cell line observed under a confocal microscope. Lysosomal marker LTR (red), cells cultured ellipticine ELLI (green), and nuclei labeled with Hoechst 33342 dye (blue). Co-localization of ellipticine and LysoTracker is shown in yellow as the overlap of these two signals (Merge). Cells were cultured either with ellipticine alone (CTRL) or in combination with bafilomycin A (BAF), which depleted lysosomal structures and led to ellipticine redistribution. Representative images from one of three independent experiments. Confocal images, TDR, magnifications 630x
